# A standardized nomenclature for mammalian histone genes

**DOI:** 10.1186/s13072-022-00467-2

**Published:** 2022-10-01

**Authors:** Ruth L. Seal, Paul Denny, Elspeth A. Bruford, Anna K. Gribkova, David Landsman, William F. Marzluff, Monica McAndrews, Anna R. Panchenko, Alexey K. Shaytan, Paul B. Talbert

**Affiliations:** 1 European Molecular Biology Laboratory, European Bioinformatics Institute, Wellcome Genome Campus, Hinxton, CB10 1SD UK; 2 Department of Haematology, School of Clinical Medicine, University of Cambridge, Cambridge, CB2 0PT UK; 3 Department of Biology, Lomonosov Moscow State University, 119234 Moscow, Russia; 4 Intramural Research Program, National Library of Medicine, National Institutes of Health, Bethesda, MD 20892 USA; 5 Integrated Program for Biological and Genome Sciences, University of North Carolina, Chapel Hill, NC 27599 USA; 6 Mouse Genome Informatics, The Jackson Laboratory, 600 Main Street, Bar Harbor, ME 04609 USA; 7 Department of Pathology and Molecular Medicine, School of Medicine, Queen’s University, Kingston, Ontario Canada; 8 Howard Hughes Medical Institute, Fred Hutchinson Cancer Research Center, 1100 Fairview Avenue N, Seattle, WA 98109 USA

## Abstract

Histones have a long history of research in a wide range of species, leaving a legacy of complex nomenclature in the literature. Community-led discussions at the EMBO Workshop on Histone Variants in 2011 resulted in agreement amongst experts on a revised systematic protein nomenclature for histones, which is based on a combination of phylogenetic classification and historical symbol usage. Human and mouse histone gene symbols previously followed a genome-centric system that was not applicable across all vertebrate species and did not reflect the systematic histone protein nomenclature. This prompted a collaboration between histone experts, the Human Genome Organization (HUGO) Gene Nomenclature Committee (HGNC) and Mouse Genomic Nomenclature Committee (MGNC) to revise human and mouse histone gene nomenclature aiming, where possible, to follow the new protein nomenclature whilst conforming to the guidelines for vertebrate gene naming. The updated nomenclature has also been applied to orthologous histone genes in chimpanzee, rhesus macaque, dog, cat, pig, horse and cattle, and can serve as a framework for naming other vertebrate histone genes in the future.

**Supplementary Information:**

The online version contains supplementary material available at 10.1186/s13072-022-00467-2.

## Introduction

The DNA in all eukaryotic cells is packaged with histones to form chromatin. The basic unit of chromatin in eukaryotes, the nucleosome, consists of 147 base pairs (bp) of DNA wrapped around an octamer of four core histones, comprising an H3–H4 tetramer and two H2A–H2B dimers. In multicellular organisms, there is a histone H1 bound to the linker region between two nucleosomes, which binds to the region where DNA enters and exits the nucleosome. In addition to packaging the DNA into the nucleus, histones play multiple roles in gene expression, DNA replication and DNA damage repair. The core histones can be extensively modified on their N- and C-terminal tails and globular domains, and these modifications may change binding sites for regulatory factors or neutralize the charge of lysine residues via acetylation. These modifications may silence genes or activate them. Not all DNA is packaged into nucleosomes, there are regions of nucleosome-free DNA, particularly at promoters and enhancers of active genes. There are multiple histone H3, H2A and H1 protein variants which replace the canonical histones at specific sites in the genome. Some of these variants are expressed throughout the cell cycle in all cells, while others are expressed predominantly in specific tissues.

Every time a cell divides, it must not only replicate its DNA but also synthesize large amounts of histones to package the newly replicated DNA. In mammalian cells this requires synthesis of about 10^8^ molecules of each of the four core histone proteins. In metazoans, these histone proteins are encoded by the set of replication-dependent histone genes, which encode representatives of all five classes of histone proteins [1]. The replication-dependent histone genes encode messenger RNAs (mRNAs) which differ from all other cellular mRNAs: instead of being polyadenylated, these mRNAs end in a stem-loop structure. These genes do not contain introns, and the only processing event is cleavage of the nascent transcript to form the 3’ end of the histone mRNA. Some of the “replication-dependent” histone genes can also produce polyadenylated mRNAs. For example, analysis of global gene expression in normal non-dividing tissues revealed that a subset of 10 human replication-dependent histone genes produced polyadenylated mRNAs in all non-dividing tissues analyzed [2].

### Genomic clustering of replication-dependent histone genes

In mammals, the replication-dependent genes are found at four discrete loci. In the human genome the largest cluster is on chromosome 6 and contains more than 60 genes, and the second cluster on chromosome 1 contains 10–12 genes. There are 4 genes in a third distinct locus on chromosome 1, and a single replication-dependent histone H4 gene on chromosome 12 (with a neighboring H2A gene for which replication dependency is uncertain). This genomic organization is conserved, and all four loci are syntenic, in mammals.

In other vertebrates, the organization of histone genes is variable. In chicken, there is a single large cluster, analogous to the largest mammalian cluster, which contains genes for all five histone types, all of which encode mRNAs ending in a stem loop. Many fish (e.g., zebrafish) and amphibians (e.g., Xenopus), as well as invertebrates like sea urchins and Drosophila, store large amounts of histone mRNA and proteins in the egg and start development with a series of rapid cell cycles in the absence of zygotic transcription. These species contain far more copies of histone genes than there are in mammals, many of which are organized in tandem repeats with one copy of all 5 histone gene types in each repeat. This is an adaptation to fulfill the requirement for synthesis of large amounts of histone mRNA and protein in a short period of time, either in oogenesis (e.g., in zebrafish, Drosophila and Xenopus), or in early embryogenesis (e.g., in sea urchins) depending on the species.

The replication-dependent histone genes in metazoans are present in a nuclear body, the histone locus body (HLB). Transcription and processing of the histone mRNA occurs within the HLB [3]. This structure provides a microenvironment in the nucleus specialized for the biosynthesis of histone mRNAs, and many factors unique to histone mRNA biosynthesis are concentrated there. The critical protein required for formation of the HLB is NPAT, which is only found bound to the replication-dependent histone genes [4]. Phosphorylation of NPAT by Cyclin E/CDK2 is essential for activation of histone gene expression as cells approach S-phase [5]. The need to form the HLB explains why genes for all five histone types involved in replication-dependent histone synthesis are clustered in the genome in metazoans [6].

### Replication-independent histones

Replication-independent variant histone genes are usually expressed throughout the cell cycle, typically contain introns, and are transcribed into polyadenylated mRNAs. These genes are positioned throughout the genome, often as single copies, and are not bound by NPAT. There is one histone gene, the *H2AX* gene, that encodes both a polyadenylated mRNA and an mRNA that ends in a stem loop, and expresses the stem-loop mRNA only in S-phase [7, 8]. Since this gene is not bound by NPAT, it is not considered a replication-dependent gene, although it does express a replication-dependent histone mRNA which is cell-cycle regulated [7]. The variety of histone proteins encoded in the mammalian genome are described in [9] and are presented below along with the corresponding protein nomenclature.

### Histone protein nomenclature

Nomenclature for histones has been an evolving topic since their discovery, with standardization of the protein names H1, H2A, H2B, H3, and H4 dating to the Ciba Foundation Symposium of 1975 [10], and revised in 2005 [11] with the addition of suffixes for post-translational modifications. The discovery of the many histone variants in the genomics era resulted in variant names using a plethora of styles, including prefixes, number suffixes, letter suffixes, a variety of punctuation types or no punctuation, synonyms, and near-homographs, resulting in a call for standardization at the EMBO Workshop on Histone Variants in 2011. The outcome of this workshop was summarized in the protein nomenclature proposed by Talbert et al. [12] in 2012, which restricted itself to naming proteins on the basis that gene nomenclature can be organism specific and that paralogous genes can encode an identical histone variant. The resulting protein nomenclature retained the designations H1, H2A, H2B, H3 and H4 for the five classes of histones, and tried to build on existing variant designations while systematizing naming principles. It aimed to balance historical usage with a phylogenetic approach to naming histone variants, using a period (.), already in use to append suffixes to variant names, to designate branch points in the phylogeny. This is referred to hereafter as the “Strasbourg nomenclature” after the location of the EMBO workshop.

### Gene nomenclature committees

The HUGO (Human Genome Organization) Nomenclature Committee (HGNC) has been in operation since the late 1970s and is the only group with the authority to approve nomenclature for human genes. The committee has recently published updated guidelines [13] with a new focus on providing stability for the clinical community. The HGNC also has a sister project, the Vertebrate Gene Nomenclature Committee (VGNC) that approves gene symbols and names for selected vertebrate species of community interest with high quality genomes (currently chimpanzee, rhesus macaque, dog, cat, horse, cattle and pig). The HGNC and VGNC both work in close coordination with the other existing nomenclature committees for model organisms, especially the Mouse Genomic Nomenclature Committee (MGNC) of the Mouse Genome Database [14]. HGNC and VGNC gene symbols are in uppercase letters, while rodent gene symbols have an initial uppercase letter followed by lowercase letters. Wherever possible, orthologous genes are assigned equivalent gene symbols across the HGNC, VGNC and MGNC. These committees do not have jurisdiction over protein nomenclature.

### Previous histone gene nomenclature

The HGNC and MGNC both previously approved gene nomenclature for the replication-dependent histone genes as described in “The human and mouse replication-dependent histone genes” [15]. This nomenclature system was based on the genomic cluster that the histone genes were located on, with each symbol beginning HIST1/Hist1 for histone cluster 1 (the largest cluster of over 60 genes located on chromosome 6 in human and chromosome 13 in mouse), HIST2/Hist2 for cluster 2 (located on chromosome 1 in human and 3 in mouse; contains 10–12 core histone genes), HIST3/Hist3 for cluster 3 (a small cluster of 4 genes that has not been well studied) and HIST4/Hist4 for “cluster 4” (the smallest cluster containing one replication-dependent H4 gene). The gene symbols then included the histone type encoded by the gene followed by a unique letter identifier, e.g., human symbol *HIST1H2AA* and mouse symbol *Hist1H2aa* represented the first (“a”) histone type H2A gene on histone cluster 1. This system had many strengths, such as providing equivalent symbols for most human and mouse orthologs within the clusters. However, those without prior knowledge of histone genes could wrongly assume that the start of the gene symbols represented the histone type. Furthermore, these symbols comprised 9 characters and feedback, including from clinical communities, often requests avoiding the designation of long symbols. Although the organization of histone gene clusters is conserved across mammals, this is not true across all vertebrates—chicken has a single large replication-dependent histone cluster [16]—also meaning that the gene symbols starting with histone cluster identifiers were not transferrable across non-mammalian vertebrate species.

Replication-independent histone variant genes followed a completely separate nomenclature system. These genes were assigned symbols that started with the histone type, then ‘F’ for family, followed by an identifying letter. The letters were usually based on the encoded histone variant as used by the community, e.g., *H2AFZ* designated the first human gene identified as encoding the H2A.Z variant [17], but the letters could also simply be assigned by the nomenclature committee, e.g., the symbols *H2AFY* and *H2AFY2* were approved for genes encoding the macroH2A subtypes [18]. Having these two separate nomenclature systems for the replication-dependent and -independent genes was arguably problematic, as this large and complex gene family was not united by a common root symbol.

## Revised histone gene nomenclature

As mentioned above, the agreed unified nomenclature for histone protein variants was published by 42 experts from the histone field [12]. The HGNC and MGNC were already aware of problems with the existing gene nomenclature, so both committees agreed that they would work towards revising histone gene symbols—with the input of histone researchers—to be as close to the unified protein nomenclature as possible, whilst fulfilling the requirements of standardized mammalian gene nomenclature. For example, one feature of the histone protein nomenclature is the use of periods as separators in symbols to indicate protein variants and/or proteins that represent phylogenetic branch points. Approved gene symbols cannot include periods, primarily as this could cause problems in data processing. It was agreed that where periods exist between a letter and number in protein variant symbols, these are left out of the new gene symbols entirely, e.g., *H2AZ1* is the symbol for the gene encoding the H2A.Z.1 variant. Where a separator is needed between two consecutive numbers, a hyphen is used for HGNC and VGNC gene symbols, and the letter ‘f’ in mouse gene symbols, e.g., genes encoding the H1.0 variant are *H1-0* in the HGNC and VGNC databases and *H1f0* for mouse. Hyphens are avoided in mouse gene symbols because punctuation is reserved for specific usage in mouse allele nomenclature.

The new gene nomenclature uses symbols that begin with the letter H followed by a numeral, or numeral and letter, to indicate which major histone type they encode, e.g., “*H2BC3*” encodes an H2B type histone. Tissue of expression as a characteristic is not used in the revised histone gene names, due to variability of reporting, discovery of expression in other tissues in new datasets, and possible lack of conservation across species. Efforts have been made to create a nomenclature that makes sense across vertebrates where possible—the revised symbols no longer refer to individual clusters, meaning that the naming scheme can be extended into non-mammalian vertebrates, e.g., human *HIST1H2AA*, “histone cluster 1 H2A family member a” has been renamed as *H2AC1*, “H2A clustered histone 1”. Replication-independent genes are named as closely to the agreed upon symbols for the protein variants as possible.

### Resolving replication-dependent histone gene nomenclature across mammalian species

**Fig. 1:**
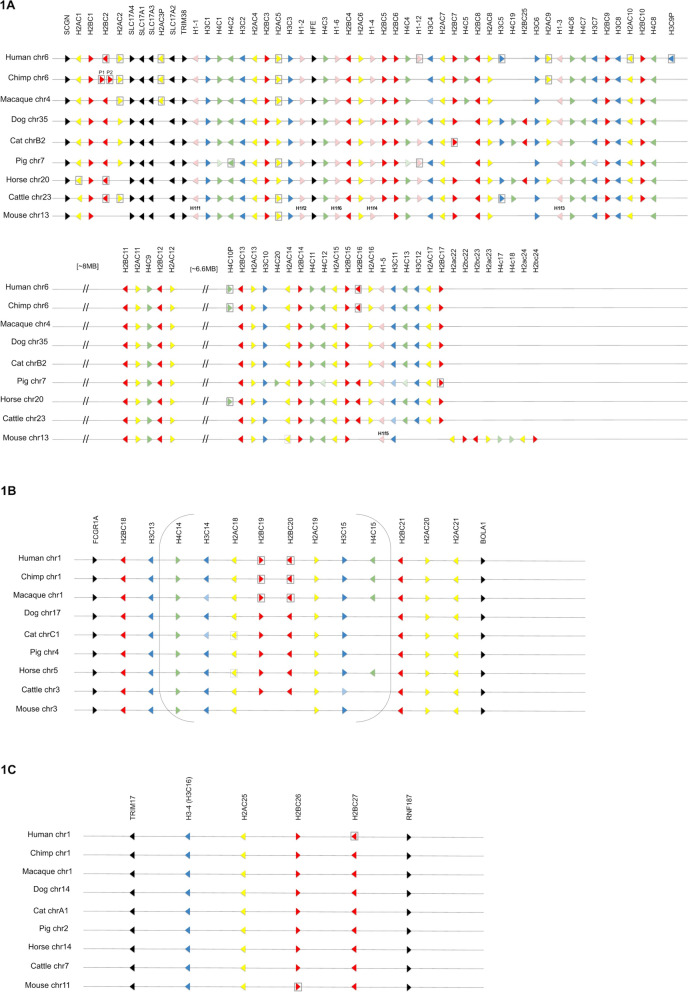
The three replication-dependent histone gene clusters in mammals. Gene symbols are shown across the top; species and chromosomal location of each cluster is indicated at the side. Black = non-histone genes, pink = histone H1 genes, yellow = H2A genes, red = H2B genes, blue = H3 genes, green = H4 genes. Pseudogenes are indicated by a gray box around the gene (pseudogenes have the same symbol as their protein-coding orthologs but end with -ps for mouse and P for the other species). A paler shade indicates that the gene is present but currently unannotated and unnamed; a blank space indicates that the gene is missing entirely. Mouse H1 genes contain an ‘f’ in place of the hyphen, so each mouse H1f symbol is shown above each relevant mouse gene. **A**: The largest replication-dependent cluster, also known as HIST1. There are two large gaps in the cluster in all species. Conservation between species is remarkable, although there are some species-specific duplications, gene losses and in situ pseudogenizations. Mouse has an expansion at the end of the cluster—these genes are shown with the mouse gene symbol format; note that all mouse symbols follow this format but for simplicity only the uppercase format used for other mammalian genes is shown for the conserved genes. **B**. The second largest replication-dependent cluster, also known as HIST2; each species has at least 10 genes in this cluster that contains genes for the 4 core histones but contains no histone H1 genes. The cluster contains a large inverted repeat, indicated by brackets. **C**. The third mammalian replication-dependent cluster, also known as HIST3. Note that *H3-4* has an exceptional symbol due to the common usage of the H3.4 symbol for the protein encoded by this gene, the systematic H3C16 alias is shown in parentheses

Histone protein nomenclature does not encompass the complexity of histone genes where many paralogs encode identical, or very similar, proteins. The distinct H2A and H2B isoforms encoded by genes within the replication-dependent clusters contain many small variations which have not been characterized as functionally significant and are not well conserved across species. There are two H3 isoforms on the largest two replication-dependent largest clusters, H3.1 and H3.2; all human H3 genes on cluster 1 encode H3.1, while some of the orthologous mouse genes on cluster 1 encode H3.2. For this reason, it is not possible to approve one gene symbol per replication-dependent protein isoform. However, due to the remarkable conservation of gene order within the mammalian replication-dependent clusters, it is possible to identify one-to-one orthologs for most of the genes and name orthologs with equivalent gene symbols (see Fig. 1).

The previous replication-dependent histone gene nomenclature was curated for human and mouse only. It is impossible for automated naming systems to resolve orthology well enough to assign gene symbols to most replication-dependent histone genes in other mammalian species. Therefore, VGNC curators have manually named histone genes in the species chimpanzee, rhesus macaque, dog, cat, pig, horse and cattle to be the same as their identified human orthologs, using a combination of conserved gene order and sequence similarity (Fig. 1 and Additional File 1). Note that numbering of genes in the clusters is not intended to reflect gene order, so that symbols for any additional paralogs identified in new species may be added, e.g., dog, cattle and horse all have the genes *H4C19* and *H2BC25* which are not present in human or mouse and therefore take higher numbers, so that the nearest 5’ H2B gene to *H2BC25* is *H2BC8* and the nearest 5’ H4 gene to *H4C19* is *H4C5* (Fig. 1A).

Comparing the histone clusters across mammalian species has resulted in the resolution of several former gene symbol differences between human and mouse orthologs. In mouse, the genes now named as *H2ac18* and *H2ac19* were previously named as *Hist2h2aa1* and *Hist2h2aa2*, while the human orthologs that are now named consistently as *H2AC18* and *H2AC19* were previously named with different symbols—*HIST2H2AA3* and *HIST2H2AA4*. The inverted repeat within histone cluster 2 was missing from the initial human reference genome, meaning that only one copy of each human gene in this repeat was included in the initial round of naming. The primate inverted repeats include two pseudogenes (*H2BC19P*, previously *HIST2H2BD* and *H2BC20P*, previously *HIST2H2BC*) that are not present in mouse. Aligning these genes with the additional species shows that in cat, pig, horse and cattle these genes encode an H2B protein and the mRNA ends in a stem loop (Fig. 1B), and therefore, these are functional genes and are named as *H2BC19* and *H2BC20* in cat, pig, horse and cattle (the ‘P’ at the end of the human symbols indicates the locus is a pseudogene). In all species, these H2BC genes are flanked by *H2AC18* and *H2AC19* (note that *H2AC18* appears to be pseudogenized in cat and horse) in a conserved gene order and gene orientation. Therefore, it is clear to see that *H2BC19* and *H2BC20* orthologs have been lost in mouse but the flanking *H2ac18* and *H2ac19* genes remain and can be named in concordance with their orthologs in other mammals (Fig. 1A).

Pseudogenization of genes in situ is a feature of replication-dependent histone clusters; studying cluster organization across other mammals has enabled us to rename more mouse and human pseudogenes to be in line with their protein-coding orthologs. Human *H2AC10P* was not previously named as orthologous to mouse—its previous symbol was *HIST1H2APS4*, while the previous symbol for the coding mouse ortholog (now *H2ac10*) was *Hist1h2af*. All other mammalian species studied here appear to have a coding copy of *H2AC10* (Fig. 1A), making it clear that the human gene at this conserved position is a pseudogenized ortholog and can be named accordingly as *H2AC10P*. As for *H2BC19P* and *H2BC20P* mentioned above, there are several other human pseudogenes that have no equivalent mouse ortholog, which have now been named relative to protein-coding orthologs in other species. For example, *H1-12* is predicted to be coding in dog, horse and cattle (although the predicted H1.12 protein has not been studied) allowing the naming of the human pseudogenized ortholog as *H1-12P* (previously *HIST1H1PS1*); pig also carries a pseudogenized copy which is again named as *H1-12P* (Fig. 1A). *H2BC2* and *H2AC2* are predicted to be coding in dog, cat and pig, resulting in the renaming of human *HIST1H2BPS1* and *HIST1H2APS1* as *H2BC2P* and *H2AC2P* (Fig. 1A).

### Full description of the revised gene nomenclature by histone type

#### H1 histone genes

The revised H1 histone gene nomenclature, along with previous symbols and protein variant symbols, is shown in Table 1 for human and Table 2 for mouse. All symbols begin with the root ‘H1’. The H1 genes were relatively simple to fit with the Strasbourg protein nomenclature, where each H1 variant is distinguished by a separate number, because each H1 gene encodes a different histone variant. It has, therefore, been possible to approve gene symbols that are equivalent to the H1 protein symbols, shown in Talbert et al. [12]. As mentioned above, HGNC and VGNC gene symbols include a hyphen in place of a period where two numbers need to be separated. The H1 nomenclature distinguishes replication-dependent from replication-independent genes in the gene names by the presence of the words “cluster member” but “C” for cluster is not used in the gene symbols to preserve parallel H1 protein and gene symbols. The terminology “cluster member” was chosen to feature at the end of replication-dependent histone H1 gene names rather than “clustered”, which is used elsewhere, so that the gene names of all H1 genes can contain the type of H1 followed by the term ‘linker histone’, e.g., “H1.1 linker histone, cluster member”. It is now clear to the non-expert that *H1-0* and *H1-1* both encode histone H1 genes. Mouse gene symbols include an ‘f’ instead of a hyphen (Table 2) because hyphens are reserved for mouse allele nomenclature where this punctuation has a specific role. For example, in the allele *Tg(tetO-H1f0)1Hzo*, the hyphen separates the promoter from the expressed gene. Although slightly different, the mouse and human symbols are clearly equivalent, e.g., *H1f1* and *H1-1*.

**Table 1:** Revised gene nomenclature for human histone H1 genes

Variant symbol	HGNC gene symbol	HGNC gene name	Previous symbol	HGNC ID	UniProt ID
H1.0	*H1-0*	H1.0 linker histone	H1F0	HGNC:4714	P07305
H1.1	*H1-1*	H1.1 linker histone, cluster member	HIST1H1A	HGNC:4715	Q02539
H1.2	*H1-2*	H1.2 linker histone, cluster member	HIST1H1C	HGNC:4716	P16403
H1.3	*H1-3*	H1.3 linker histone, cluster member	HIST1H1D	HGNC:4717	P16402
H1.4	*H1-4*	H1.4 linker histone, cluster member	HIST1H1E	HGNC:4718	P10412
H1.5	*H1-5*	H1.5 linker histone, cluster member	HIST1H1B	HGNC:4719	P16401
H1.6	*H1-6*	H1.6 linker histone, cluster member	HIST1H1T	HGNC:4720	P22492
H1.7	*H1-7*	H1.7 linker histone	H1FNT	HGNC:24893	Q75WM6
H1.8	*H1-8*	H1.8 linker histone	H1FOO	HGNC:18463	Q8IZA3
NA	*H1-9P*	H1.9 linker histone, pseudogene	HILS1	HGNC:30616	P60008
H1.10	*H1-10*	H1.10 linker histone	H1FX	HGNC:4722	Q92522
NA	*H1-12P*	H1.12 linker histone, cluster member pseudogene	HIST1H1PS1	HGNC:19163	NA

**Table 2:** Revised gene nomenclature for mouse histone H1 genes

Variant symbol	Mouse gene symbol	Mouse gene name	Previous symbol	MGI ID	UniProt ID
H1.0	*H1f0*	H1.0 linker histone	H1f0	MGI:95893	P10922
H1.1	*H1f1*	H1.1 linker histone, cluster member	Hist1h1a	MGI:1931523	P43275
H1.2	*H1f2*	H1.2 linker histone, cluster member	Hist1h1c	MGI:1931526	P15864
H1.3	*H1f3*	H1.3 linker histone, cluster member	Hist1h1d	MGI:107502	P43277
H1.4	*H1f4*	H1.4 linker histone, cluster member	Hist1h1e	MGI:1931527	P43274
H1.5	*H1f5*	H1.5 linker histone, cluster member	Hist1h1b	MGI:1861461	P43276
H1.6	*H1f6*	H1.6 linker histone, cluster member	Hist1h1t	MGI:1888530	Q07133
H1.7	*H1f7*	H1.7 linker histone	H1fnt	MGI:117319	Q8CJI4
H1.8	*H1f8*	H1.8 linker histone	H1foo	MGI:2176207	Q8VIK3
H1.9	*H1f9*	H1.9 linker histone	Hils1	MGI:2136691	Q9QYL0
H1.10	*H1f10*	H1.10 linker histone	H1fx	MGI:2685307	Q80ZM5
NA	*H1f11-ps*	H1.11 linker histone, pseudogene	Gm6970	MGI:3645322	NA

Note that replication-dependent histone H1 genes have “cluster member” in the gene name

#### H2A histone genes

##### Replication-dependent H2A genes

The complete set of revised H2A histone genes is shown in Table 3 for human and Table 4 for mouse. Two general classes of H2A proteins were first identified by Fred Zweidler using triton-acid urea gel electrophoresis [19] based on a characteristic change at position 51 of the protein sequence where there is a leucine in H2A.1 and a methionine in H2A.2. H2A.1 and H2A.2 are encoded by multiple replication-dependent H2A histone genes and these variant designations were recommended in the unified histone nomenclature [12]. For the most part, H2A genes on the largest replication-dependent cluster, known as HIST1 (Fig. 1A), encode H2A.1 proteins, while those on the second largest cluster, known as HIST2 (Fig. 1B), encode H2A.2 proteins. The H2A protein on the smaller cluster known as HIST3 (Fig. 1C) encodes an H2A.1 protein with more amino acid changes elsewhere in the protein compared to other H2A.1-encoding genes. Human *H2AC21* on cluster 2 encodes an H2A.1 protein, while the mouse ortholog, *H2ac21*, encodes an H2A.2 protein. Therefore, the revised gene nomenclature does not distinguish between H2A.1 or H2A.2 to allow consistent naming of orthologs across vertebrate species. There are alternative histone protein naming systems that do not distinguish between H2A.1 and H2A.2 but refer to replication-dependent H2A as ‘canonical H2A’ [20]. During discussions with the wider histone community, advice was given to avoid use of the term ‘canonical’ as this term can be interpreted in different ways by different researchers. It was during this feedback process that the suggestion was made to use ‘clustered’ to refer to H2A, H2B, H3 and H4 replication-dependent genes. The H2A histone genes on replication-dependent clusters have, therefore, been named with the root symbol ‘H2AC#’ (H2ac# in mouse) for ‘H2A clustered histone’. Although there are multiple proteins encoded by the mammalian replication-dependent H2A genes, with small differences primarily at the C terminus [15], these variations are not conserved between mouse and human orthologs, suggesting they are not functional, and are therefore not reflected at the level of gene nomenclature. The *H2AC1* and *H2BC1* genes encode the H2A and H2B proteins with the largest number of amino acid changes; they also were initially reported as “sperm specific”, which likely accounts for the variability from the other genes.

**Table 3:** Revised gene nomenclature for human histone H2A genes

Variant symbol	HGNC gene symbol	HGNC gene name	Previous symbol	HGNC ID	UniProt ID
H2A	*H2AC1*	H2A clustered histone 1	HIST1H2AA	HGNC:18729	Q96QV6
NA	*H2AC2P*	H2A clustered histone 2, pseudogene	HIST1H2APS1	HGNC:18720	NA
NA	*H2AC3P*	H2A clustered histone 3, pseudogene	HIST1H2APS2	HGNC:18804	NA
H2A	*H2AC4*	H2A clustered histone 4	HIST1H2AB	HGNC:4734	P04908
NA	*H2AC5P*	H2A clustered histone 5, pseudogene	HIST1H2APS5	HGNC:4728	NA
H2A	*H2AC6*	H2A clustered histone 6	HIST1H2AC	HGNC:4733	Q93077
H2A	*H2AC7*	H2A clustered histone 7	HIST1H2AD	HGNC:4729	P20671
H2A	*H2AC8*	H2A clustered histone 8	HIST1H2AE	HGNC:4724	P04908
NA	*H2AC9P*	H2A clustered histone 9, pseudogene	HIST1H2APS3	HGNC:18805	NA
NA	*H2AC10P*	H2A clustered histone 10, pseudogene	HIST1H2APS4	HGNC:4732	NA
H2A	*H2AC11*	H2A clustered histone 11	HIST1H2AG	HGNC:4737	P0C0S8
H2A	*H2AC12*	H2A clustered histone 12	HIST1H2AH	HGNC:13671	Q96KK5
H2A	*H2AC13*	H2A clustered histone 13	HIST1H2AI	HGNC:4725	P0C0S8
H2A	*H2AC14*	H2A clustered histone 14	HIST1H2AJ	HGNC:4727	Q99878
H2A	*H2AC15*	H2A clustered histone 15	HIST1H2AK	HGNC:4726	P0C0S8
H2A	*H2AC16*	H2A clustered histone 16	HIST1H2AL	HGNC:4730	P0C0S8
H2A	*H2AC17*	H2A clustered histone 17	HIST1H2AM	HGNC:4735	P0C0S8
H2A	*H2AC18*	H2A clustered histone 18	HIST2H2AA3	HGNC:4736	Q6FI13
H2A	*H2AC19*	H2A clustered histone 19	HIST2H2AA4	HGNC:29668	Q6FI13
H2A	*H2AC20*	H2A clustered histone 20	HIST2H2AC	HGNC:4738	Q16777
H2A	*H2AC21*	H2A clustered histone 21	HIST2H2AB	HGNC:20508	Q8IUE6
H2A	*H2AC25*	H2A clustered histone 25	HIST3H2A	HGNC:20507	Q7L7L0
H2A.Z.1	*H2AZ1*	H2A.Z variant histone 1	H2AFZ	HGNC:4741	P0C0S5
H2A.Z.2	*H2AZ2*	H2A.Z variant histone 2	H2AFV	HGNC:20664	Q71UI9
macroH2A.1	*MACROH2A1*	macroH2A.1 histone	H2AFY	HGNC:4740	O75367
macroH2A.2	*MACROH2A2*	macroH2A.2 histone	H2AFY2	HGNC:14453	Q9P0M6
H2A.X	*H2AX*	H2A.X variant histone	H2AFX	HGNC:4739	P16104
H2A.J	*H2AJ*	H2A.J histone	H2AFJ	HGNC:14456	Q9BTM1
H2A.B	*H2AB1*	H2A.B variant histone 1	H2AFB1	HGNC:22516	P0C5Y9
H2A.B	*H2AB2*	H2A.B variant histone 2	H2AFB2	HGNC:18298	P0C5Z0
H2A.B	*H2AB3*	H2A.B variant histone 3	H2AFB3	HGNC:14455	P0C5Z0
H2A.P	*H2AP*	H2A.P histone	HYPM	HGNC:18417	O75409
NA	*H2AQ1P*	H2A.Q variant histone 1, pseudogene	NA	HGNC:53962	NA
H2A.L	*H2AL1Q*	H2A.L variant histone 1Q	NA	HGNC:53959	NA
NA	*H2AL1MP*	H2A.L variant histone 1 M, pseudogene	NA	HGNC:53961	NA
H2A.L	*H2AL3*	H2A.L variant histone 3	NA	HGNC:53960	NA

**Table 4:** Revised gene nomenclature for mouse histone H2A genes

Variant symbol	Mouse gene symbol	Mouse gene name	Previous symbol	MGI ID	UniProt ID
H2A	*H2ac1*	H2A clustered histone 1	Hist1h2aa	MGI:2448285	Q8CGP4
H2A	*H2ac4*	H2A clustered histone 4	Hist1h2ab	MGI:2448306	C0HKE1
NA	*H2ac5-ps*	H2A clustered histone 5, pseudogene	Gm11336	MGI:3651860	NA
H2A	*H2ac6*	H2A clustered histone 6	Hist1h2ac	MGI:2448287	C0HKE2
H2A	*H2ac7*	H2A clustered histone 7	Hist1h2ad	MGI:2448289	C0HKE3
H2A	*H2ac8*	H2A clustered histone 8	Hist1h2ae	MGI:2448290	C0HKE4
H2A	*H2ac10*	H2A clustered histone 10	Hist1h2af	MGI:2448309	Q8CGP5
H2A	*H2ac11*	H2A clustered histone 11	Hist1h2ag	MGI:2448293	C0HKE5
H2A	*H2ac12*	H2A clustered histone 12	Hist1h2ah	MGI:2448295	Q8CGP6
H2A	*H2ac13*	H2A clustered histone 13	Hist1h2ai	MGI:248457	C0HKE6
NA	*H2ac14-ps*	H2A clustered histone 14, pseudogene	Hist1h2aj	MGI:2448312	NA
H2A	*H2ac15*	H2A clustered histone 15	Hist1h2ak	MGI:2448297	Q8CGP7
H2A	*H2ac18*	H2A clustered histone 18	Hist2h2aa1	MGI:96097	Q6GSS7
H2A	*H2ac19*	H2A clustered histone 19	Hist2h2aa2	MGI:2448283	Q6GSS7
H2A	*H2ac20*	H2A clustered histone 20	Hist2h2ac	MGI:2448316	Q64523
H2A	*H2ac21*	H2A clustered histone 21	Hist2h2ab	MGI:2448314	Q64522
H2A	*H2ac22*	H2A clustered histone 22	Hist1h2an	MGI:2448300	C0HKE7
H2A	*H2ac23*	H2A clustered histone 23	Hist1h2ao	MGI:2448302	C0HKE8
H2A	*H2ac24*	H2A clustered histone 24	Hist1h2ap	MGI:3710573	C0HKE9
H2A	*H2ac25*	H2A clustered histone 25	Hist3h2a	MGI:2448458	Q8BFU2
H2A.Z.1	*H2az1*	H2A.Z variant histone 1	H2afz	MGI:1888388	P0C0S6
H2A.Z.2	*H2az2*	H2A.Z variant histone 2	H2afv	MGI:1924855	Q3THW5
macroH2A.1	*Macroh2a1*	macroH2A.1 histone	H2afy	MGI:1349392	Q9QZQ8
macroH2A.2	*Macroh2a2*	macroH2A.2 histone	H2afy2	MGI:3037658	Q8CCK0
H2A.X	*H2ax*	H2A.X variant histone	H2afx	MGI:102688	P27661
H2A.J	*H2aj*	H2A.J histone	H2afj	MGI:3606192	Q8R1M2
H2A.B	*H2ab1*	H2A.B variant histone 1	H2ab1	MGI:3642445	S4R1E0
H2A.B	*H2ab2*	H2A.B variant histone 2	H2ab2	MGI:3644980	S4R1M3
H2A.B	*H2ab3*	H2A.B variant histone 3	H2ab3	MGI:3644875	S4R1G7
H2A.P	*H2ap*	H2A.P histone	Hypm	MGI:1914584	Q9CR04
H2A.L	*H2al1a*	H2A histone family member L1A	NA	MGI:3714114	Q5M8Q2
H2A.L	*H2al1b*	H2A histone family member L1B	NA	MGI:3650131	A0A087WP11
H2A.L	*H2al1c*	H2A histone family member L1C	NA	MGI:3711280	Q5M8Q2
H2A.L	*H2al1d*	H2A histone family member L1D	NA	MGI:3710419	Q5M8Q2
H2A.L	*H2al1e*	H2A histone family member L1E	NA	MGI:3649617	Q810S6
H2A.L	*H2al1f*	H2A histone family member L1F	NA	MGI:3649874	Q5M8Q2
H2A.L	*H2al1g*	H2A histone family member L1G	NA	MGI:3710577	Q5M8Q2
H2A.L	*H2al1h*	H2A histone family member L1H	NA	MGI:3711282	Q5M8Q2
H2A.L	*H2al1i*	H2A histone family member L1I	NA	MGI:3710416	Q5M8Q2
H2A.L	*H2al1j*	H2A histone family member L1J	NA	MGI:3643273	A2BFR3
H2A.L	*H2al1k*	H2A histone family member L1K	NA	MGI:3710586	J3QP08
H2A.L	*H2al1m*	H2A histone family member L1M	NA	MGI:1923633	Q9DAD9
H2A.L	*H2al1n*	H2A histone family member L1N	NA	MGI:3643774	Q497L1
H2A.L	*H2al1o*	H2A histone family member L1O	NA	MGI:3643069	L7MU04
NA	*H2al1q-ps*	H2A histone family member L1Q, pseudogene	NA	MGI:3705686	NA
NA	*H2al1r-ps*	H2A histone family member L1R, pseudogene	NA	MGI:3705677	NA
H2A.L	*H2al2a*	H2A histone family member L2A	NA	MGI:1915481	Q9CQ70
H2A.L	*H2al2b*	H2A histone family member L2B	NA	MGI:3710623	A9Z055
H2A.L	*H2al2c*	H2A histone family member L2C	NA	MGI:3779546	A9Z055
H2A.L	*H2al3*	H2A histone family member L3	NA	MGI:1922521	Q9D4U4

##### Replication-independent H2A genes

For replication-independent H2A histones the protein nomenclature has been followed as closely as possible. For example, the human genes encoding the H2A.Z variant that is present in all eukaryotes [21] have been approved as *H2AZ1* and *H2AZ2* (full gene names “H2A.Z histone 1” and “H2A.Z histone 2”). The macroH2A variant was so named because it is almost three times as large as replication-dependent H2A histones [22]. This variant name is included in the Strasbourg nomenclature and accepted by the histone community. Therefore, an exception has been made and the corresponding gene symbols *MACROH2A1* and *MACROH2A2* (*MacroH2a1* and *MacroH2a2* for mouse) approved, even though this means that the symbols do not begin with the root symbol ‘H2A’.

The Strasbourg nomenclature aims to “use letter suffixes for monophyletic clades” [12]. However, the experts that devised this nomenclature recognized that in some cases historical usage and community support for such usage should be taken into consideration. Therefore, they recommended that the histone community continue to use the H2A.X designation even though this histone variant does not appear to be from a separate clade to the replication-dependent H2A histones that have been assigned the root symbol H2AC. This recommendation has been followed and the gene named as *H2AX*; this gene is not positioned within a replication-dependent cluster and is interesting as it encodes two distinct mRNAs, one ending in a stem loop and the other one polyadenylated [8]. The stem-loop form of the mRNA is expressed in S-phase of the cell cycle and the polyadenylated form expressed outside of S-phase. The *H2AX* gene does not bind NPAT, distinguishing it from the genes in the clusters.

The same principle has been followed for the gene named as *H2AJ*, which also does not belong to a separate clade to the replication-dependent H2A histone genes; the encoded variant has been published as H2A.J [23] and has been referred to as replication independent [9]. For the species reported here, the gene produces only polyadenylated mRNA, and does not contain a stem loop; hence, this has been assigned the separate variant-type symbol *H2AJ*. Note the *H2AJ* gene is adjacent to the *H4C16* gene.

##### Short H2A replication-independent variants

Short histone H2A variants lack a C-terminal region compared to replication-dependent histones and all appear to be expressed primarily in the testis, although H2A.B is also expressed in brain [24]. These variants all derive from a single gene on the X chromosome of a common ancestor and have since diverged into four distinct clades known as H2A.P, H2A.Q, H2A.B, and H2A.L [25]. The H2A.P-encoding gene had the previously approved symbol *HYPM* for “huntingtin interacting protein M” and has now been renamed as *H2AP* for “H2A.P histone” (and from *Hypm* to *H2ap* in mouse). H2A.Q is the most recently discovered short H2A variant [25] and a functional protein has been predicted for many non-Euarchontoglires mammals. However, the only VGNC species with a supporting protein-coding gene annotation is dog; this gene has been named as *H2AQ1,* for “H2A.Q variant histone 1” (Additional File 1). In human the locus is pseudogenized at a conserved position on the X chromosome and has been named *H2AQ1P* for “H2A.Q variant histone 1, pseudogene”. Although the presence of an orthologous mouse pseudogene is suggested in [25], there is currently no annotated mouse gene.

Human and most other mammals have three paralogs that encode H2A.B histones. In humans, these duplicated paralogs neighbor coagulation factor VIII genes and are numbered consistently with these genes—*H2AB1* is next to *F8A1*; *H2AB2* is next to *F8A2*; *H2AB3* is next to *F8A3*. All three H2AB genes are highly similar in sequence and encode a protein that is identical in the case of *H2AB2* and *H2AB3*, with only one amino acid difference in the protein encoded by *H2AB1*. In the literature these two proteins have sometimes been referred to as the variants H2A.B.1 (encoded by *H2AB2* and *H2AB3*) and H2A.B.2 (encoded by *H2AB1*) [25], although many papers do not make this distinction and refer to variant H2A.B only [26–28]. Mouse has three paralogs named *H2ab1*, *H2ab2* and *H2ab3*; the mouse-encoded H2A.B protein has been referred to as H2A.B.3 [29].

Mouse has an expansion of H2A.L-encoding genes, with fourteen H2al1 protein-coding genes (named *H2al1a* through to *H2al1o*), three H2al2 genes (*H2al2a*, *H2al2b* and *H2al2c*) which are the only H2al family members to be found outside of the X chromosome, and one H2al3 gene. Although no H2A.L protein has been detected in human so far [25], human has an ortholog of mouse *H2al3* with an intact open reading frame which has therefore been named *H2AL3*. This gene is conserved in rhesus macaque, cattle, pig, horse and dog (Additional File 1). There is also a human H2AL1 family member, *H2AL1Q*, which has an intact open reading frame, so has the potential to encode a protein. There is a mouse pseudogene, *H2al1q-ps*, at a syntenic location and this locus is predicted to be coding in dog, cat and cattle (Additional File 1). Finally, there is a human gene at a conserved genomic position to mouse *H2al1m*, but this is a pseudogene and has therefore been named *H2AL1MP*.

#### Histone H2B genes

Revised human H2B gene nomenclature is shown in Table 5; revised mouse H2B gene nomenclature is shown in Table 6. In accordance with the H2A genes on replication-dependent clusters described above, H2B genes on these clusters have been named with the root symbol H2BC# for ‘H2B clustered histone’ (H2bc# in mouse). Attempts have been made to follow the Strasbourg nomenclature as closely as possible for the H2B replication-independent genes.

**Table 5:** Revised gene nomenclature for human histone H2B genes

Variant symbol	HGNC gene symbol	HGNC gene name	Previous symbol	HGNC ID	UniProt ID
H2B	*H2BC1*	H2B clustered histone 1	HIST1H2BA	HGNC:18730	Q96A08
NA	*H2BC2P*	H2B clustered histone 2, pseudogene	HIST1H2BPS1	HGNC:18719	NA
H2B	*H2BC3*	H2B clustered histone 3	HIST1H2BB	HGNC:4751	P33778
H2B	*H2BC4*	H2B clustered histone 4	HIST1H2BC	HGNC:4757	P62807
H2B	*H2BC5*	H2B clustered histone 5	HIST1H2BD	HGNC:4747	P58876
H2B	*H2BC6*	H2B clustered histone 6	HIST1H2BE	HGNC:4753	P62807
H2B	*H2BC7*	H2B clustered histone 7	HIST1H2BF	HGNC:4752	P62807
H2B	*H2BC8*	H2B clustered histone 8	HIST1H2BG	HGNC:4746	P62807
H2B	*H2BC9*	H2B clustered histone 9	HIST1H2BH	HGNC:4755	Q93079
H2B	*H2BC10*	H2B clustered histone 10	HIST1H2BI	HGNC:4756	P62807
H2B	*H2BC11*	H2B clustered histone 11	HIST1H2BJ	HGNC:4761	P06899
H2B	*H2BC12*	H2B clustered histone 12	HIST1H2BK	HGNC:13954	O60814
H2B	*H2BC13*	H2B clustered histone 13	HIST1H2BL	HGNC:4748	Q99880
H2B	*H2BC14*	H2B clustered histone 14	HIST1H2BM	HGNC:4750	Q99879
H2B	*H2BC15*	H2B clustered histone 15	HIST1H2BN	HGNC:4749	Q99877
NA	*H2BC16P*	H2B clustered histone 16, pseudogene	HIST1H2BPS2	HGNC:4754	NA
H2B	*H2BC17*	H2B clustered histone 17	HIST1H2BO	HGNC:4758	P23527
H2B	*H2BC18*	H2B clustered histone 18	HIST2H2BF	HGNC:24700	Q5QNW6
NA	*H2BC19P*	H2B clustered histone 19, pseudogene	HIST2H2BD	HGNC:20517	Q6DRA6
NA	*H2BC20P*	H2B clustered histone 20, pseudogene	HIST2H2BC	HGNC:20516	Q6DN03
H2B	*H2BC21*	H2B clustered histone 21	HIST2H2BE	HGNC:4760	Q16778
H2B	*H2BC26*	H2B clustered histone 26	HIST3H2BB	HGNC:20514	Q8N257
NA	*H2BC27P*	H2B clustered histone 27, pseudogene	HIST3H2BA	HGNC:20515	NA
H2B.K	*H2BK1*	H2B.K variant histone 1	H2BE1	HGNC:53833	A0A2R8Y619
NA	*H2BL1P*	H2B.L histone variant 1, pseudogene	H2BP4	HGNC:54442	NA
H2B.W	*H2BW1*	H2B.W histone 1	H2BFWT	HGNC:27252	Q7Z2G1
H2B.W	*H2BW2*	H2B.W histone 2	H2BFM	HGNC:27867	P0C1H6
NA	*H2BW3P*	H2B.W histone 3, pseudogene	NA	HGNC:44390	NA
NA	*H2BW4P*	H2B.W histone 4, pseudogene	H2BFXP	HGNC:25757	NA
H2B.N	*H2BN1*	H2B.N variant histone 1	NA	HGNC:56200	NA
H2B	*H2BC12L*	H2B clustered histone 12 like	H2BFS	HGNC:4762	P57053

**Table 6:** Revised gene nomenclature for mouse histone H2B genes

Variant symbol	Mouse gene symbol	Mouse gene name	Previous symbol	MGI ID	UniProt ID
H2B	*H2bc1*	H2B clustered histone 1	Hist1h2ba	MGI:2448375	P70696
H2B	*H2bc3*	H2B clustered histone 3	Hist1h2bb	MGI:2448377	Q64475
H2B	*H2bc4*	H2B clustered histone 4	Hist1H2bc	MGI:1915274	Q6ZWY9
H2B	*H2bc6*	H2B clustered histone 6	Hist1h2be	MGI:2448380	Q6ZWY9
H2B	*H2bc7*	H2B clustered histone 7	Hist1h2bf	MGI:2448383	P10853
H2B	*H2bc8*	H2B clustered histone 8	Hist1h2bg	MGI:2448386	Q6ZWY9
H2B	*H2bc9*	H2B clustered histone 9	Hist1h2bh	MGI:2448387	Q64478
H2B	*H2bc11*	H2B clustered histone 11	Hist1h2bj	MGI:2448388	P10853
H2B	*H2bc12*	H2B clustered histone 12	Hist1h2bk	MGI:2448399	Q8CGP1
H2B	*H2bc13*	H2B clustered histone 13	Hist1h2bl	MGI:2448403	P10853
H2B	*H2bc14*	H2B clustered histone 14	Hist1h2bm	MGI:2448404	P10854
H2B	*H2bc15*	H2B clustered histone 15	Hist1h2bn	MGI:2448407	P10853
H2B	*H2bc18*	H2B clustered histone 18	Hist2h2bb	MGI:2448413	Q64525
H2B*	*H2bc21*	H2B clustered histone 21	Hist2h2be	MGI:2448415	Q64524
H2B	*H2bc22*	H2B clustered histone 22	Hist1h2bp	MGI:2448409	Q8CGP2
H2B	*H2bc23*	H2B clustered histone 23	Hist1h2bq	MGI:3702051	Q8CBB6
H2B	*H2bc24*	H2B clustered histone 24	Hist1h2br	MGI:3710645	Q8CBB6
NA	*H2bc26-ps*	H2B clustered histone 26, pseudogene	Hist3h2bb-ps	MGI:1922442	NA
H2B	*H2bc27*	H2B clustered histone 27	Hist3h2ba	MGI:1925553	Q9D2U9
SubH2Bv/H2B.L**	*H2bl1*	H2B.L histone variant 1	1700024P04Rik	MGI:1916632	Q9D9Z7
H2B.W	*H2bw2*	H2B.W histone 2	H2bfm	MGI:1916639	Q9DAB5

^*^This variant has never been characterized experimentally and so has no Strasbourg variant name

^**^This variant has been published with two different symbols; following community consultation this gene has been named as encoding the H2B.L variant

##### H2B.W-encoding histone genes

The H2B.W variant symbol was proposed in the Strasbourg nomenclature [12] for the variant that had been previously known as H2BFWT [30, 31] and TH2B-175 [31]. Human has two H2B.W-encoding paralogs which are now named as *H2BW1* and *H2BW2* and two pseudogenes (*H2BW3P* and *H2BW4P*) all located on the X chromosome between *RAB9B* and *SLC25A3*, while mouse has only one H2B.W-encoding gene (*H2bw2*) found in a syntenic location. Other mammals have between 1 and 4 H2BW paralogs but these are all located at the same conserved location of the X chromosome (Additional File 1).

##### H2B.L-encoding histone genes

Another mammalian H2B variant was first published as SubH2Bv [32] based on its location in the subacrosomal component of cattle spermatozoa. The homologous mouse variant was published as H2BL1 (originally to denote H2B-like 1) [33]. For the macroH2A variant mentioned above, an exception was made, and the MACROH2A# gene symbols were approved due to the overwhelming usage of macroH2A in the scientific literature. In contrast, the SubH2Bv/H2BL variant has not been well published. Following discussions between the HGNC and groups that have published on this variant, it was agreed to use H2BL# for the genes encoding this variant so that the root symbol H2B# is preserved. Therefore, the cattle gene is now named *H2BL1* for “H2B.L histone” (Additional File 1), and the mouse ortholog has the equivalent symbol *H2bl1*. Human has a pseudogenized version of this gene, which is therefore named as *H2BL1P*, “H2B.L histone variant 1, pseudogene”.

##### H2B.K-encoding histone genes

The *H2BK1* gene was first discovered via gene annotation [34] and was independently identified in a recent study on H2B variants [35]. There is no mouse ortholog of this gene, but there are one-to-one orthologs in many other mammals (including all curated VGNC species, see Additional File 1), birds and fish. In human there are transcripts overlapping *H2BK1* and the upstream gene *ABCF2*, which, combined with the lack of mouse ortholog, had previously meant this histone gene was not annotated. According to Hidden Markov Model classification using the ‘Analyze sequence’ tool at the HistoneDB 2.0 database [36], the encoded protein does not match a characterized histone variant (67% identity with the most similar protein encoded by the other H2B genes). Therefore, at the initial time of naming, it was decided to name this gene as encoding a new histone variant. This gene was originally approved as *H2BE1* for “H2B.E variant histone 1” but as H2B.E has been used in the literature several times for an isoform of mouse *H2bc21*, the nomenclature has been updated to avoid possible confusion. The variant identifier H2B.K was agreed with the authors of [35] ahead of their publication and the gene has been updated with the corresponding gene symbol *H2BK1* and name “H2B.K variant histone 1”.

##### H2B.N-encoding histone genes

The *H2BN1* gene encodes the most recently described H2B variant, H2B.N [35]. Like the *H2BK1* gene, the human *H2BN1* gene has an exon overlapping a separate gene, in this case the long non-coding RNA gene *MYO1D-DT*, and has no protein-coding ortholog in mouse. Additionally, as noted in [35], the *H2BN1* and *H2BK1* genes are both composed of two exons with the same part of the coding sequence encoding the histone fold domain split by the intron in both genes, but phylogenetic analysis in [35] does not support a common origin for the two variants. The *H2BK1* gene is present in mammals, fish, birds and reptiles while *H2BN1* is only found in mammals. It should be noted that neither the protein variant H2B.K nor H2B.N has been experimentally determined.

##### H2BC12L histone gene

There is a human-specific duplication of the *H2BC12* gene from the chromosome 6 replication-dependent cluster gene on chromosome 21. CAGE tag data [37] supports expression of this gene and as there are no frameshifts or deletions within the open reading frame, it is annotated as coding. Although the gene appears to be expressed there is no direct evidence that a protein is produced. The encoded protein does not represent a new histone variant—it only has one nonsynonymous amino acid difference from the H2B protein encoded by the parent gene *H2BC12*, and is classified as a “canonical” histone when analyzing the sequence via the Histone DB2.0 database. Therefore, this gene has been named as *H2BC12L* for “H2B clustered histone 12 like”.

#### Histone H3 genes

##### Replication-dependent H3 genes

Nomenclature for histone H3 genes for human is shown in Table 7 and for mouse in Table 8. Histone H3 genes on major replication-dependent clusters are named with the root symbol ‘H3C#’ for ‘H3 clustered histone’ (H3c# in mouse). The Strasbourg nomenclature refers to histone H3.1 and H3.2 for histone proteins encoded on the larger replication-coupled clusters. However, as for H2A.1 and H2A.2 above, it has not been possible to reflect this in the gene nomenclature as it would not allow for consistent naming across orthologs. The H3.1 and H3.2 proteins are identical except that H3.1 has a cysteine at position 96 while H3.2 has a serine at this position. Again, there are examples where an ortholog in one species may encode an H3.1 protein and an H3.2 protein in another, e.g., human *H3C2* encodes an H3.1 protein while mouse *H3c2* encodes an H3.2 protein. Therefore, the H3.1 vs H3.2 distinction is not reflected in the gene nomenclature.

**Table 7:** Revised gene nomenclature for human histone H3 genes

Variant symbol	HGNC gene symbol	HGNC gene name	Previous symbol	HGNC ID	UniProt ID
H3.1	*H3C1*	H3 clustered histone 1	HIST1H3A	HGNC:4766	P68431
H3.1	*H3C2*	H3 clustered histone 2	HIST1H3B	HGNC:4776	P68431
H3.1	*H3C3*	H3 clustered histone 3	HIST1H3C	HGNC:4768	P68431
H3.1	*H3C4*	H3 clustered histone 4	HIST1H3D	HGNC:4767	P68431
NA	*H3C5P*	H3 clustered histone 5, pseudogene	NA	HGNC:54427	NA
H3.1	*H3C6*	H3 clustered histone 6	HIST1H3E	HGNC:4769	P68431
H3.1	*H3C7*	H3 clustered histone 7	HIST1H3F	HGNC:4773	P68431
H3.1	*H3C8*	H3 clustered histone 8	HIST1H3G	HGNC:4772	P68431
NA	*H3C9P*	H3 clustered histone 9, pseudogene	HIST1H3PS1	HGNC:18982	NA
H3.1	*H3C10*	H3 clustered histone 10	HIST1H3H	HGNC:4775	P68431
H3.1	*H3C11*	H3 clustered histone 11	HIST1H3I	HGNC:4771	P68431
H3.1	*H3C12*	H3 clustered histone 12	HIST1H3J	HGNC:4774	P68431
H3.2	*H3C13*	H3 clustered histone 13	HIST2H3D	HGNC:25311	Q71DI3
H3.2	*H3C14*	H3 clustered histone 14	HIST2H3C	HGNC:20503	Q71DI3
H3.2	*H3C15*	H3 clustered histone 15	HIST2H3A	HGNC:20505	Q71DI3
H3.3	*H3-3A*	H3.3 histone A	H3F3, H3F3A	HGNC:4764	P84243
H3.3	*H3-3B*	H3.3 histone B	H3F3B	HGNC:4765	P84243
H3.4	*H3-4*	H3.4 histone, cluster member	HIST3H3	HGNC:4778	Q16695
H3.5	*H3-5*	H3.5 histone	H3F3C	HGNC:33164	Q6NXT2
NA (H3.6)*	*H3P16*	H3 histone pseudogene 16	H3F3AP6	HGNC:42982	NA
H3.7**	*H3-7*	H3.7 histone (putative)	HIST2H3PS2	HGNC:32060	Q5TEC6
NA (H3.8)***	*H3P44*	H3 histone pseudogene 44	H3F3AP5	HGNC:42981	NA
H3.Y.1	*H3Y1*	H3.Y histone 1	NA	HGNC:43735	P0DPK2
H3.Y.2	*H3Y2*	H3.Y histone 2	NA	HGNC:43734	P0DPK5
cenH3	*CENPA*	centromere protein A	NA	HGNC:1851	P49450

^*^The variant symbol is shown as NA (H3.6) because the encoding gene is annotated as a pseudogene and, therefore, named within the H3 pseudogene series as *H3P16*, but the variant H3.6 has been reported in the literature

^**^Although this histone variant has been referred to as H3.7 in the literature, its existence is in doubt and it was not included in the Strasbourg variant nomenclature

^***^The variant symbol is shown as NA (H3.8) because, as for *H3P16* above, the encoding gene is annotated as a pseudogene, named as *H3P44*, but the variant H3.8 has been reported in the literature

**Table 8:** Revised gene nomenclature for mouse histone H3 genes

Variant symbol	Mouse gene symbol	Mouse gene name	Previous symbol	MGI ID	UniProt ID
H3.1	*H3c1*	H3 clustered histone 1	Hist1h3a	MGI:2668828	P68433
H3.2	*H3c2*	H3 clustered histone 2	Hist1h3b	MGI:2448319	P84228
H3.2	*H3c3*	H3 clustered histone 3	Hist1h3c	MGI:2448320	P84228
H3.2	*H3c4*	H3 clustered histone 4	Hist1h3d	MGI:2448322	P84228
H3.2	*H3c6*	H3 clustered histone 6	Hist1h3e	MGI:2448326	P84228
H3.2	*H3c7*	H3 clustered histone 7	Hist1h3f	MGI:2448329	P84228
H3.1	*H3c8*	H3 clustered histone 8	Hist1h3g	MGI:2145541	P68433
H3.1	*H3c10*	H3 clustered histone 10	Hist1h3h	MGI:2448349	P68433
H3.1	*H3c11*	H3 clustered histone 11	Hist1h3i	MGI:2448350	P68433
H3.2	*H3c13*	H3 clustered histone 13	Hist2h3b	MGI:2448351	P84228
H3.2	*H3c14*	H3 clustered histone 14	Hist2h3c1	MGI:2448355	P84228
H3.2	*H3c15*	H3 clustered histone 15	Hist2h3c2	MGI:2448357	P84228
H3.3	*H3f3a*	H3.3 histone A	H3f3a	MGI:1097686	P84244
H3.3	*H3f3b*	H3.3 histone B	H3f3b	MGI:1101768	P84244
H3.4	*H3f4*	H3.4 histone, cluster member	Gm12260	MGI:3651326	NA
cenH3	*Cenpa*	centromere protein A	NA	MGI:88375	O35216

##### Replication-independent H3 genes

The H3.3 replication-independent variant is found across metazoa [38] while the H3.4 variant is found only in mammals [39]. The H3.3 variant is encoded by two mammalian genes which have been named as *H3-3A* and *H3-3B* for “H3.3 histone A” and “H3.3 histone B” (*H3f3a* and *H3f3b* in mouse). The H3.4 variant is encoded by a gene previously named *HIST3H3* in human and the uninformative gene symbol *Gm12260* in mouse. This variant is also commonly referred to as H3.1t [39] or H3t [40] because it was originally thought to be testis specific, but it has since been shown to be expressed at lower levels in other tissues [41]. The Strasbourg nomenclature recommendation was to refer to this as histone H3.4 which supports the symbol first published for this variant [42]. The H3.4 protein has a conserved valine residue at position 25 which has been reported to affect binding of the N-terminal tail by the Tudor domain of PHF1 and PHF19 [41, 43]. Due to the referral in the literature of this as an H3 variant, and the ‘H3.4’ recommendation by the Strasbourg nomenclature, we have named this gene *H3-4* in human and other VGNC species (*H3f4* in mouse). The gene encodes mRNA with a stem-loop structure and is adjacent to genes named with the H2AC# and H2BC# root symbols (*H2AC25* and *H2BC26*). To reflect its position on a replication-dependent cluster, we have given this gene the full name “H3.4 histone, cluster member” and have added the gene symbol alias “H3C16” (see Fig. 1C).

Following discussions with the histone community, the symbol *CENPA* has been retained for the H3-like histone encoding gene that is found at the nucleosome core of centromeric chromatin [44], but the symbol alias “cenH3” has been included for this gene.

#### Primate-specific predicted H3 variants

This section describes symbols and names for a number of primate histone H3 genes. Note that it is not trivial to decide whether histone duplications limited to individual species, or even orders, are protein coding or should be represented as pseudogenes. Other mammals may also have additional predicted protein-coding histone genes that have not yet been named because these species are not currently supported by manual annotation projects.

##### H3.5-encoding histone gene

The H3.5 variant is a hominid-specific testis expressed gene that is likely a duplication of the *H3-3B* gene via retrotransposition [45]. While the *H3-3A* and *H3-3B* genes encode the same H3.3 protein, the protein predicted from the duplication is distinct. For this reason, common usage in the scientific literature and the variant identifier mentioned in the Strasbourg nomenclature have been followed and the gene named *H3-5* for “H3.5 histone”.

##### H3.7-encoding histone gene

The H3.7 variant identified in [46] is encoded by a duplication of the *H3C13* gene, located roughly 6 MB upstream of the “cluster 2” replication-dependent histone genes. The predicted protein is most like an H3.2 variant, but H3.2 variants are characterized by a serine at residue 96 while the H3.7 variant has an arginine at residue 96, which is not seen in any other H3 histone proteins. The nomenclature published in [46] has been followed and the gene assigned as *H3-7* with the gene name “H3.7 histone (putative)”. The term “putative” will be removed if there is future experimental evidence that this variant exists. Taguchi et al. [46] also identified two further putative H3 variants which they called H3.6 and H3.8. However, there are insufficient expression data to support annotation of these genes as protein coding, so in the absence of further data, the encoding genes are annotated and named as H3 pseudogenes: *H3P16* (H3 histone pseudogene 16) and *H3P44* (H3 histone pseudogene 44). These pseudogenes have been given the aliases H3.6 and H3.8.

##### H3.Y-encoding histone genes

The H3.Y variant is encoded by two genes in human, which were initially referred to as H3.X and H3.Y [47]. As H3.Y forms a clear primate-specific clade incorporating both genes, and the protein referred to as ‘H3.X’ is only predicted from mRNA sequence, the recommendations of the Strasbourg nomenclature have been followed and the two human genes assigned as *H3Y1* and *H3Y2* for “H3.Y histone 1” and “H3.Y histone 2”. While human and chimpanzee have two paralogs, rhesus macaque appears to only have *H3Y1* (Additional File 1). However, in chimp a symbol has only been approved for the *H3Y2* ortholog (Additional File 1) because “*H3Y1*” is currently on an unplaced scaffold; updates to the chimpanzee genome may result in the putative chimp “*H3Y1*” being assigned.

#### Histone H4 genes

The genes encoding histone H4 proteins are mostly found within replication-dependent clusters and are, thus, named with the root symbol H4C# for “H4 clustered histone” for human (Table 9) and H4c# for mouse (Table 10). All human H4 genes encode the same protein except for *H4C7*, which encodes an H4 protein with a truncated C terminus [9], sometimes referred to as H4.7 or H4.G [9]. Interestingly, there is no mouse *H4C7* ortholog, although an ortholog is present in chimp, dog, pig, horse and cattle, only the chimp ortholog appears to encode the same H4.7/H4.G variant as human, while the *H4C7* gene in dog, pig, horse and cattle encodes the same H4 protein as other H4 genes. A study [48] of the putative protein encoded by human *H4C7* found that it is expressed at low levels and in vitro forms unstable nucleosomes. The *H4C7* gene is annotated as protein coding because several unique peptides from proteomic projects map to the *H4C7* locus on the human genome reference GRCh38; however, as this gene has only been reported to be expressed in tumor cells, its existence as a true histone variant in normal cells is in doubt.

**Table 9:** Revised gene nomenclature for human histone H4 genes

Variant symbol	HGNC gene symbol	HGNC gene name	Previous symbol	HGNC ID	UniProt ID
H4	*H4C1*	H4 clustered histone 1	HIST1H4A	HGNC:4781	P62805
H4	*H4C2*	H4 clustered histone 2	HIST1H4B	HGNC:4789	P62805
H4	*H4C3*	H4 clustered histone 3	HIST1H4C	HGNC:4787	P62805
H4	*H4C4*	H4 clustered histone 4	HIST1H4D	HGNC:4782	P62805
H4	*H4C5*	H4 clustered histone 5	HIST1H4E	HGNC:4790	P62805
H4	*H4C6*	H4 clustered histone 6	HIST1H4F	HGNC:4783	P62805
H4*	*H4C7*	H4 clustered histone 7	HIST1H4G	HGNC:4792	Q99525
H4	*H4C8*	H4 clustered histone 8	HIST1H4H	HGNC:4788	P62805
H4	*H4C9*	H4 clustered histone 9	HIST1H4I	HGNC:4793	P62805
NA	*H4C10P*	H4 clustered histone 10, pseudogene	HIST1H4PS1	HGNC:4786	NA
H4	*H4C11*	H4 clustered histone 11	HIST1H4J	HGNC:4785	P62805
H4	*H4C12*	H4 clustered histone 12	HIST1H4K	HGNC:4784	P62805
H4	*H4C13*	H4 clustered histone 13	HIST1H4L	HGNC:4791	P62805
H4	*H4C14*	H4 clustered histone 14	HIST2H4A	HGNC:4794	P62805
H4	*H4C15*	H4 clustered histone 15	HIST2H4B	HGNC:29,607	P62805
H4	*H4C16*	H4 histone 16	HIST4H4	HGNC:20,510	P62805

^*^ This protein predicted to be encoded by the human *H4C7* gene has been referred to in the literature as H4.7 or H4.G but its existence is in doubt and there is no separate symbol in the Strasbourg unified nomenclature

**Table 10:** Revised gene nomenclature for mouse histone H4 genes

Variant symbol	Mouse gene symbol	Mouse gene name	Previous symbol	MGI ID	UniProt ID
H4	*H4c1*	H4 clustered histone 1	Hist1h4a	MGI:2448419	P62806
H4	*H4c2*	H4 clustered histone 2	Hist1h4b	MGI:2448420	P62806
H4	*H4c3*	H4 clustered histone 3	Hist1h4c	MGI:2448421	P62806
H4	*H4c4*	H4 clustered histone 4	Hist1h4d	MGI:2448423	P62806
H4	*H4c6*	H4 clustered histone 6	Hist1h4f	MGI:2448425	P62806
H4	*H4c8*	H4 clustered histone 8	Hist1h4h	MGI:2448427	P62806
H4	*H4c9*	H4 clustered histone 9	Hist1h4i	MGI:2448432	P62806
H4	*H4c11*	H4 clustered histone 11	Hist1h4j	MGI:2448436	P62806
H4	*H4c12*	H4 clustered histone 12	Hist1h4k	MGI:2448439	P62806
H4	*H4c14*	H4 clustered histone 14	Hist2h4	MGI:2140113	P62806
H4	*H4c16*	H4 histone 16	Hist4h4	MGI:2448443	P62806
H4	*H4c17*	H4 clustered histone 17	Hist1h4m	MGI:2448441	P62806
H4	*H4c18*	H4 clustered histone 18	Hist1h4n	MGI:4843992	P62806

*H4C16* is the only gene outside of the large mammalian replication-dependent clusters and encodes the same protein as the other H4C genes. In human and other mammals, the *H4C16* and *H2AJ* genes are neighboring and are flanked by the genes *WBP11*, *SMCO3* and *ART4* while intriguingly in chicken *ART4*, *SMCO3* and *WBP11* are at the 5' end of the single large chicken histone gene cluster. The *H4C16* gene is single exon, encodes an mRNA with a stem loop and encodes a protein identical to other H4C genes. It is expressed at high levels in all cells examined, is cell-cycle regulated like the replication-dependent genes in larger clusters, and is bound by the factor NPAT which is only bound to the promoters of replication-dependent histone genes ([7]; [4]). Therefore, this gene has been assigned as *H4C16* with the gene name “H4 histone 16”. This is in contrast to the neighboring *H2AJ* gene, which has been published as a separate variant to other H2A genes, encodes an mRNA with a polyadenylated tail, and has been reported to be expressed in senescent rather than replicating cells [23].

### Adoption of the new nomenclature by the Histone Sequence Database

Wherever possible, the HGNC encourages specialist resources to display approved gene symbols to help disseminate nomenclature to the research community. The Histone Sequence Database was first introduced in the mid-1990s with the aim of maintaining a comprehensive collection of all known histone protein sequences in different species [20]. The current version of the database has extended this effort by attributing every histone sequence to a specific histone type (H3, H4, H2A, H2B, H1), histone variant (e.g., H2A.Z, H3.Y, subH2B, etc.), or canonical histone groups including variant-specific annotations [36]. The current version of the database holds 79948 histone sequences with a taxonomic span of 3160 species. The histone database can be used to explore the diversity of histone proteins and their sequence variants in many organisms to better understand how sequence variation may affect functional and structural features of nucleosomes, to browse the histone phylogenetic trees and examine variant-specific features.

Based on the updated list of human histone genes described above a comprehensive list of human histone proteins was compiled, grouped into specific histone types and histone variants. For a few histone variant genes, the attribution to a certain histone variant class has not yet been clearly established in the literature; these include *H3P16*, *H3-7, H3P44 and H4C7*. The resulting table consists of 130 entries and is provided as an Additional File (Additional File 2). The list of histone proteins has been integrated into the current version of the Histone Sequence Database (“HistoneDB 2.0: a histone database with variants”) [49] and is available at https://histdb.intbio.org/human/. The interactive table provides links to the pages of HistoneDB 2.0, the HGNC website, the Ensembl database [50], the NCBI RefSeq database [51] and PubMed.

## Conclusions

We have approved a standardized nomenclature for the complex multigene histone family that can be applied across vertebrate species. The nomenclature follows agreed upon conventions for histone protein nomenclature as closely as possible. The revised gene symbols are shorter and group histone genes together based on the type of encoded histone protein. The full human and mouse histone gene nomenclature is presented in this paper and data for chimpanzee, rhesus macaque, dog, cat, pig, horse and cattle are shown in the additional files. We encourage the histone community to reference these gene symbols in all future publications on mammalian histone genes.

## Supplementary Information

**Additional file 1. **Gene nomenclature for chimpanzee, rhesus macaque, dog, cat, pig, horse and cattle histone genes. For each gene, the VGNC ID, gene symbol and gene name are provided. Where applicable, the HGNC ID for each orthologous human gene is listed. Full Symbol Reports for each gene can be accessed using these IDs at https://vertebrate.genenames.org/.

**Additional file 2. **List of human histone proteins from the Histone Sequence Database for every histone gene, the available set of transcript and coding sequence GENCODE annotations were obtained from the Ensembl 105 database. Next, only protein-coding transcripts identical between Ensembl automated annotation and HAVANA manual curation were retained. In those cases where several transcripts of one gene correspond to the same amino acid protein sequence, only one record was retained with preference given to those that match NCBI’s RefSeq annotation. For every histone gene the list includes the HGNC symbol, information about corresponding protein sequences, their length, accession numbers within NCBI and Ensembl resources, as well as a list of relevant literature references in the form of PubMed identifiers. For a few histone variant genes, the attribution to a certain histone variant class has not yet been clearly established in the literature; these are marked in the list by a question mark.

## Data Availability

All human gene nomenclature can be fully accessed and downloaded from https://www.genenames.org/. All mouse gene nomenclature can be fully accessed and downloaded from http://www.informatics.jax.org/. All chimpanzee, rhesus macaque, dog, cat, cattle, horse and pig gene nomenclature can be fully accessed and downloaded from https://vertebrate.genenames.org/. HistoneDB 2.0 data can be fully accessed and downloaded from https://histdb.intbio.org.
